# First-principles study of defect chemistry and thermoelectric performance of CaMg_2_Sb_2_

**DOI:** 10.1039/d6ra03855g

**Published:** 2026-07-02

**Authors:** Shuai Zhang, Yuan Liu, Wenjing Qiu, Juan Li, Qidong Wang, Kai Han, Xiaole Qiu, Bing Sun

**Affiliations:** a School of Physics and Electronic Information, Shandong Key Laboratory of Gallium Nitride Materials and Applications, Weifang University Weifang 261061 China lj_wfu@163.com 20170005@wfu.edu.cn; b Jinan Key Laboratory of X-ray Optics Jinan 250000 China; c Key Laboratory of Low-Dimensional Structural Physics and Application, Education Department of Guangxi Zhuang Autonomous Region, College of Physics and Electronic Information Engineering, Guilin University of Technology Guilin 541004 China

## Abstract

The success of high-performance n-type Mg_3_Sb_2_ has sparked interest in n-type thermoelectric transport of other AM_2_X_2_ compounds. However, only a few such compounds have realized n-type transport so far, and their *zT* values remain low. Therefore, there is an urgent need to systematically understand the factors limiting their n-type thermoelectric performance. Here, using first-principles calculations, this work systematically investigates the defect chemistry, electronic structure, and thermoelectric properties of three-dimensional (3D) bulk CaMg_2_Sb_2_. Calculated results show that the n-type system shows a much higher power factor than the p-type counterpart, reaching 59 µW cm^−1^ K^−1^ at 300 K and 39 µW cm^−1^ K^−1^ at 725 K, and exhibits a maximum *zT* of ∼1.52 at 725 K. Defect analysis reveals that Sb-rich conditions favor the formation of Ca vacancies, while Mg-rich and Ca-rich conditions favor the formation of Mg and Ca interstitials, yielding n-type conduction with an electron concentration of 3 × 10^16^ cm^−3^ at 725 K. These findings suggest that establishing Mg-rich and Ca-rich growth conditions is one of the key factors for achieving high-performance n-type thermoelectric transport. This study is expected to provide theoretical guidance for achieving high-performance n-type CaMg_2_Sb_2_ thermoelectric materials.

## Abstract 2

Compared with p-type transport, n-type CaMg_2_Sb_2_ is predicted to exhibit a maximum *zT* of ∼1.52 at 725 K, with the Ca-rich and Mg-rich growth conditions favoring its transport performance.

## Graphical abstract

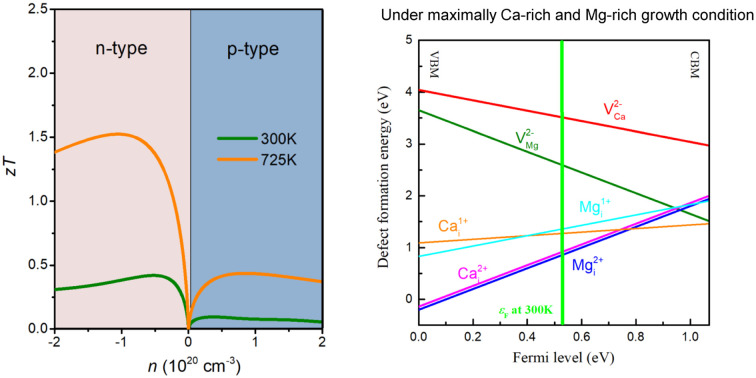

## 1. Introduction

The ever-increasing global energy crisis and environmental concerns have driven the search for clean and efficient energy conversion technologies. Thermoelectric materials,^1,2^ which can enable direct and reversible conversion between heat and electricity, exhibit great promise for waste heat recovery and solid-state refrigeration. The conversion efficiency of a thermoelectric material is governed by the dimensionless Figure of Merit, *zT* = *S*^2^*σT*/*κ*, where *S*, *σ*, *κ*, and *T* represent the Seebeck coefficient, electrical conductivity, total thermal conductivity, and absolute temperature, respectively.^3,4^ A high *zT* requires a large power factor (*S*^2^*σ*) and a low thermal conductivity. However, these transport parameters are strongly coupled with each other, making it challenging to enhance *zT* independently.^5^ Therefore, exploring and developing novel thermoelectric materials with decoupled transport properties has become a key strategy to overcome the current performance bottleneck.

The ideal candidate materials should exhibit “phonon-glass electron-crystal” (PGEC) behavior, meaning they simultaneously possess excellent electrical transport properties and extremely low thermal conductivity. Zintl phase thermoelectric materials naturally exhibit this characteristic.^6–8^ In Zintl phases, the electropositive atoms donate electrons to the electronegative atoms, with the latter forming covalent anionic frameworks for stability. The coexistence of ionic and covalent bonds in this unique structure enables high carrier mobility and low lattice thermal conductivity. Among them, the AM_2_X_2_ family of compounds is a typical class of Zintl thermoelectric materials.^9,10^ In this family, the A site is usually occupied by divalent alkaline earth metals (Mg, Ca, Sr, Ba) or rare earth metals (Yb, Eu, Sm, *etc.*), the M site is usually occupied by transition metals or divalent metals (such as Mg, Mn, Zn, Cd, *etc.*), and the X site is occupied by group V anions (N, P, As, Sb, Bi). Its crystal structure is composed of alternately stacked layers of A cations and M_2_X_2_ anionic slabs. These compounds are intrinsically p-type thermoelectric materials.^11–15^ The successful realization of high-performance n-type thermoelectricity in Mg_3_Sb_2_ has sparked extensive interest in exploring the broader n-type thermoelectric potential of this family.^14,16,17^ However, achieving high-performance n-type thermoelectric transports for other compounds remains challenging. To the best of our knowledge, apart from Mg_3_Sb_2_, which has achieved an exceptional *zT* exceeding 1.5, most compounds in this family have not yet achieved n-type transport, and only a few compounds have obtained n-type transport with relatively low performance.^16–20^

Given the extremely low n-type *zT* values of most AM_2_X_2_ compounds, a systematic understanding of the factors limiting their n-type thermoelectric performance is urgently needed. First-principles calculations have proven to be a powerful tool for predicting the thermoelectric properties of materials and revealing their defect characteristics. Among these compounds, CaMg_2_Sb_2_ compound is a promising candidate due to its threefold conduction band degeneracy, intrinsically low lattice thermal conductivity, as well as earth-abundant constituents.^11,21,22^ Although several studies^21–23^ have reported its relationship between electronic structure and electrical transport, a comprehensive investigation of the defect chemistry, electronic structure, and thermoelectric transport properties of this compound is still lacking. In this work, taking CaMg_2_Sb_2_ as an example, we systematically study the formation energies of intrinsic point defects, electron and phonon structure, and thermoelectric performance of this system using first-principles calculations. Our aim is to elucidate the intrinsic defect mechanisms governing its carrier concentration and to provide theoretical predictions for its n-type thermoelectric transport properties. The results of this study are expected to provide insights for the design of more high-performance n-type AM_2_X_2_ thermoelectric materials.

## 2. Computational methods

Electronic structure calculations are performed based on density functional theory (DFT) combined with the projected augmented wave (PAW) method as implemented in the Vienna *Ab initio* Simulation Package (VASP) package.^24–26^ The primitive cell geometry of CaMg_2_Sb_2_, including lattice constants and internal coordinates, is fully optimized using the HSE06 functional.^27^ Based on the optimized structure, the Tran–Blaha modified Becke–Johnson (TB-mBJ) potential^28,29^ is employed for electronic structure calculations to obtain accurate band gap. To investigate the effect of spin–orbit coupling (SOC), electronic structure calculations are performed both with and without SOC. The elastic constants are calculated by using finite differences method. In calculations, a 12 × 12 × 8 Monkhorst–Pack *k* mesh, a plane-wave cutoff energy of 400 eV, and an energy convergence criterion of 10^−4^ eV are used.

Electronic transport properties are derived from the calculated electronic structures using the semi-classical Boltzmann transport theory, as implemented in the BoltzTraP2 code.^30^ To evaluate electrical conductivity, the relaxation time (*τ*) is estimated by the deformation potential (DP) theory.^31^ In contrast, the temperature and carrier concentration dependence of Seebeck coefficient can obtained directly without any adjustable parameters. Lattice thermal conductivity (*k*_lat_) is calculated using a combination of the VASP and ShengBTE codes. Second-order force constant is calculated based on density functional perturbation theory (DFPT) through Phonopy code.^32^ Based on the calculated second-order force constant, the phonon spectrum is then obtained using Phonopy code. The third-order force constant is calculated based on finite displacement method through the Thirdorder code, considering at least 10 nearest neighbors to ensure computational convergence. The 6 × 6 × 4 and 1 × 1 × 1 Monkhorst–Pack *k* meshes are applied during calculating second-order and third-order interatomic force constants, respectively, and a 2 × 2 × 2 supercell is used for both calculations. Based on the obtained second-order and third-order interatomic force constants, the phonon Boltzmann transport equation is solved with the ShengBTE code to obtain lattice thermal conductivity.

To sufficiently minimize periodic image interactions of the point defects, a 3 × 3 × 2 supercell of CaMg_2_Sb_2_ containing 90 atoms is used for defect calculations, as implemented in the VASP package. During defect structure optimization, the lattice parameters are fixed at the values obtained from fully relaxed pure system using the HSE06 hybrid functional, while atomic positions are allowed to relax to their equilibrium positions. The HSE06 hybrid functional is adopted to accurately describe the energetics of point defects. A 2 × 2 × 2 Monkhorst–Pack *k* mesh is used for the supercell, and a 6 × 6 × 4 mesh is applied for the primitive cell. A plane-wave cutoff energy of 400 eV and an energy convergence criterion of 10^−4^ eV are applied.

## 3. Results and discussion

**Fig. 1:**
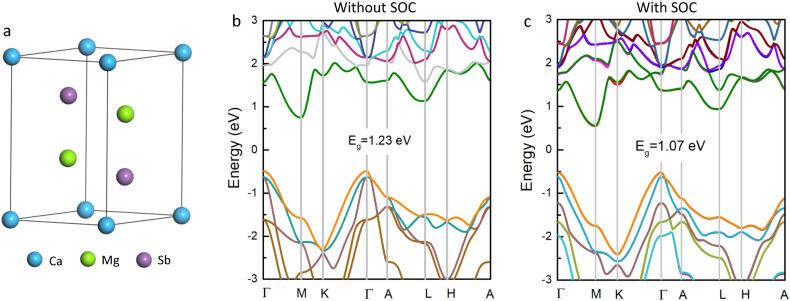
(a) Crystal structure and band structure of CaMg_2_Sb_2_ calculated (b) without and (c) with SOC.

The crystal structure of CaMg_2_Sb_2_ is shown in Fig. 1a. It crystallizes in the trigonal CaAl_2_Si_2_-structure type with space group *P*3̄*m*1. The initial atomic coordinates of the structure are directly obtained from the Inorganic Crystal Structure Database (ICSD). The optimized lattice constants by the HSE06 hybrid functional are *a* = *b* = 4.65 Å and *c* = 7.55 Å, which are consistent with the experimentally reported values (*a* = 4.65 Å, *c* = 7.58 Å) and previously reported theoretical calculation results (*a* = 4.69 Å, *c*/*a* = 1.62).^12,21^Fig. 1b and c show the calculated band structures with and without SOC, respectively. The system exhibits an indirect band gap, with the valence band maximum located at the *Γ* high-symmetry point and the conduction band minimum at the M high-symmetry point. The indirect band gap calculated with SOC is 1.07 eV, which is smaller than the value of 1.23 eV calculated without SOC. Compared to the conduction band minimum, the degeneracy of valence band maximum is significantly affected by SOC.

**Fig. 2:**
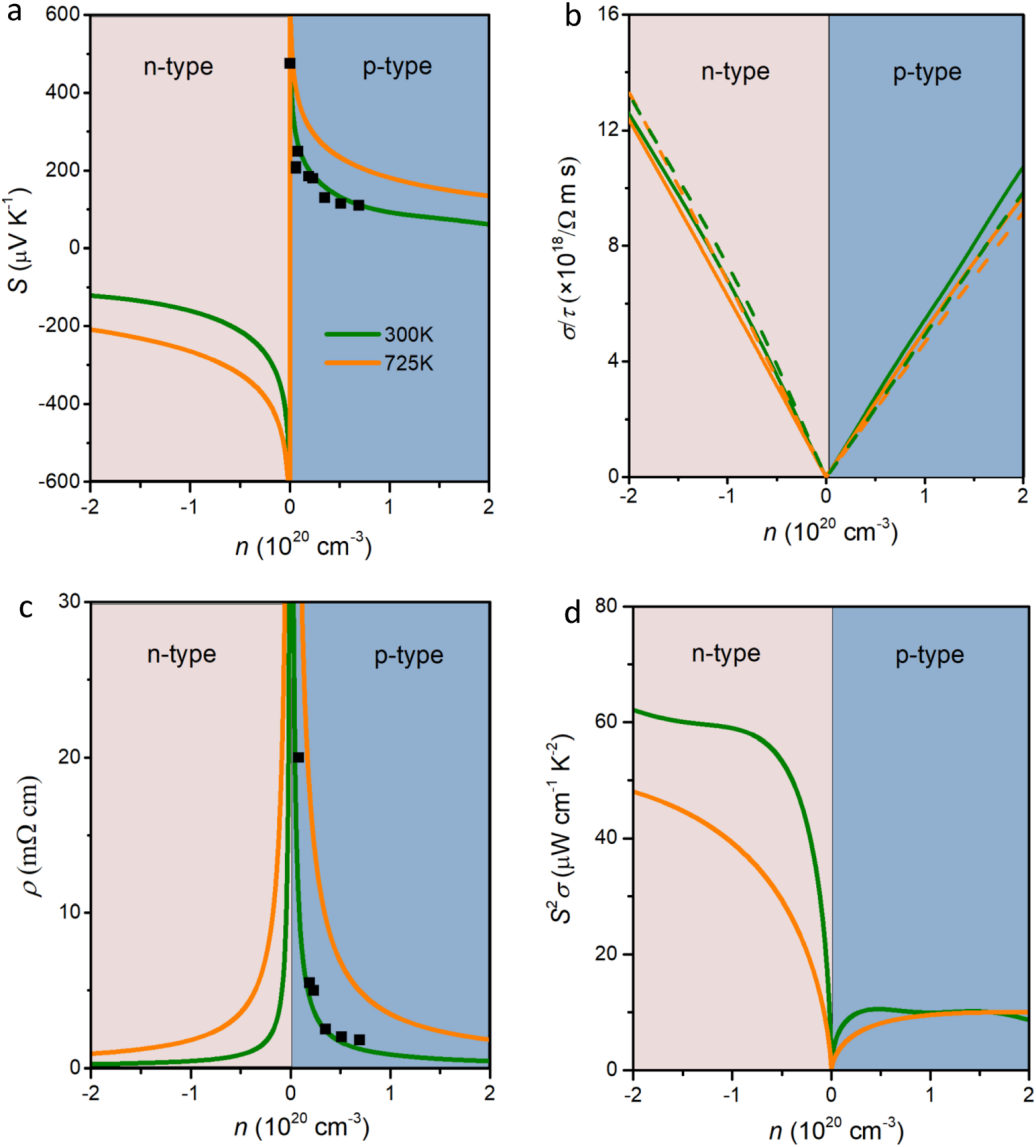
(a) Absolute Seebeck coefficient, (b) electrical conductivity/relaxation time ratio, (c) electrical resistivity, and (d) power factor of CaMg_2_Sb_2_ as a function of carrier concentration, respectively at 300 K (green curve) and 725 K **(**orange curve). The black squares represent the experimental data of p-type CaMg_2_Sb_2_ reported in the literature.^11^

Given the influence of SOC on the band structure, the band structure calculated with SOC is employed for simulating electrical transports. Fig. 2 presents the simulated p-type and n-type electrical transport properties of the system as a function of carrier concentration at temperatures of 300 K and 725 K, respectively. As shown in Fig. 2a, the Seebeck coefficient for p-type transport is positive, while that for n-type transport is negative. In both cases, the absolute value of the Seebeck coefficient decreases with increasing carrier concentration. This trend is consistent with the Pisarenko relation *S* ∝ *n*^−2/3^,^33^ reflecting the reduced average entropy per carrier at higher doping levels. The Seebeck coefficient simulated at 300 K agrees with the experimental values reported in the literature, demonstrating the reliability of the calculation method. The results of the electrical conductivity divided by relaxation time (*σ/τ*) are presented in Fig. 2b, where the values increase with increasing carrier concentration. To obtain the electrical conductivity, the relaxation time are estimated by using the deformation potential theory (see SI for computational details). The calculated average lattice elastic constant is 76.8 GPa. The calculated deformation potential constants (*Ξ*) for valence band and conduction band are 16.6 eV and 13.7 eV, respectively. By using Effective Mass Calculator (EMC) program,^34^ the effective masses of the valence band maximum are estimated to be 
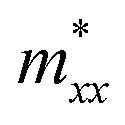
 = 1.177 m_e_, 
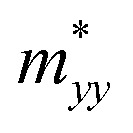
 = 1.174 m_e_, 
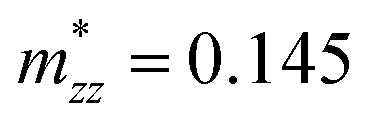
 m_e_, which yield the single valley effective mass of the valence band 
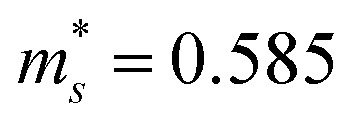
 m_e_, and the effective masses of conduction band minimum are calculated to be 
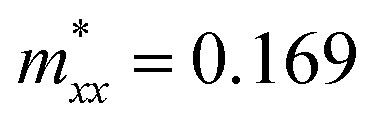
 m_e_, 
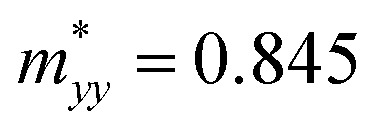
 m_e_, 
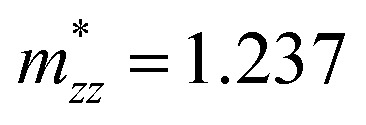
 m_e_, leading to the single valley effective mass of the conduction band 
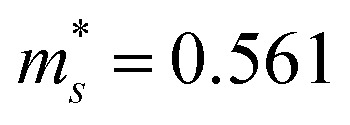
 m_e_. At 300 K and 725 K, the estimated *τ* are 2.14 × 10^−14^ s and 5.69 × 10^−15^ s for p-type transports, and 3.34 × 10^−14^ s and 8.89 × 10^−15^ s for n-type transports, respectively. Based on the estimated relaxation time, the relationship between electrical resistivity and carrier concentration is obtained, as shown in Fig. 2c. Compared to the reported experimental data, the slight discrepancy of electrical resistivity simulated at 300 K may be ascribed to the neglect of other carrier scattering mechanisms. This may lead to the overestimated *zT* values in the following calculations. Compared to p-type transport, n-type transport exhibits a higher power factor. At a carrier concentration of 1 × 10^20^ cm^−3^, the power factor for n-type transport reaches 59 µW cm^−1^ K^−1^ at 300 K and 39 µW cm^−1^ K^−1^ at 725 K.

**Fig. 3:**
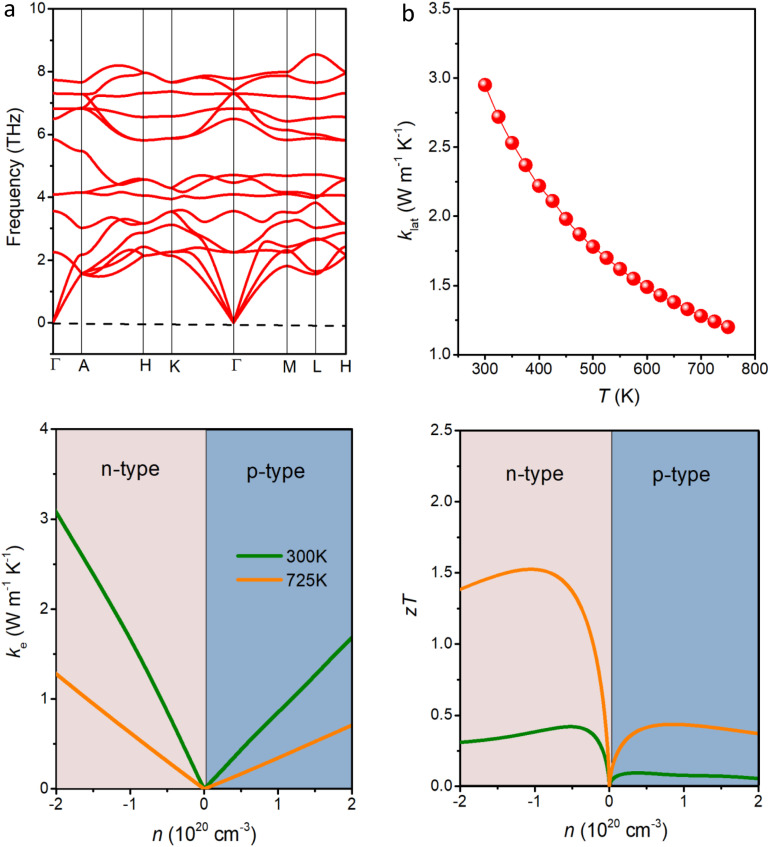
(a) Phonon spectrum, (b) temperature-dependent lattice thermal conductivity, carrier concentration dependences of (c) electronic thermal conductivity and (d) *zT* for CaMg_2_Sb_2_.

Fig. 3a shows the simulated phonon spectrum of the system. It can be seen from the figure that there are no imaginary frequencies, indicating that the structure is stable. Fig. 3b displays the simulated lattice thermal conductivity as a function of temperature. It decreases with the increase of temperature. At 300 K and 725 K, the values are 2.95 W m^−1^ K^−1^ and 1.24 W m^−1^ K^−1^, respectively. The total thermal conductivity consists of lattice thermal conductivity and electronic thermal conductivity. Based on the calculated Seebeck coefficient, the electronic thermal conductivity (*κ*_e_) is calculated according to the formula *κ*_e_ = *LT/ρ*,where *L* is the Lorentz constant calculated using the single parabolic band model (SPB) with acoustic phonon scattering.^35^ The dependence of electronic thermal conductivity on carrier concentration at temperatures of 300 K and 725 K is presented in Fig. 3c. The total thermal conductivity can be derived by combining the lattice thermal conductivity and the electronic thermal conductivity. Based on the obtained power factor and total thermal conductivity, the relationship between the *zT* and carrier concentration is predicted and shown in Fig. 3d. Compared to the p-type case, the material shows significantly better n-type thermoelectric performance, reaching a peak *zT* of 1.52 at 725 K with a carrier concentration of ∼1 × 10^20^ cm^−3^. Based on the Cahill's formula,^36,37^ the minimum lattice thermal conductivity (*κ*_min_) is calculated to be 0.58 W m^−1^ K^−1^ (see SI for computational details). This indicates that there exists potential room to further reduce the lattice thermal conductivity, which would be beneficial for further enhancing the *zT* value.

**Fig. 4:**
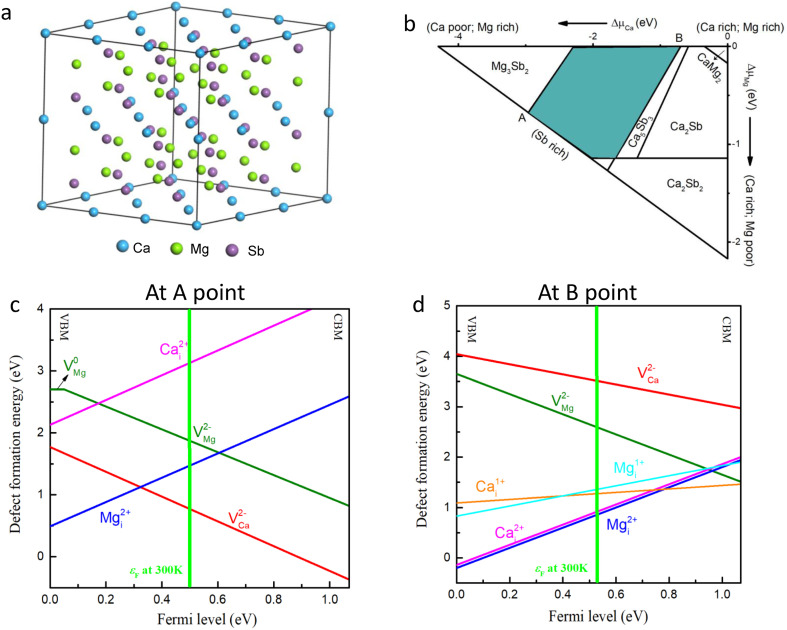
Defect chemistry of CaMg_2_Sb_2_ system: (a) a 3 × 3 × 2 supercell, (b) allowed equilibrium chemical potential region in Δ*µ*_Mg_ and Δ*µ*_Ca_ plane, shown as the shaded areas. The white regions are areas which are excluded due to the formation of competing phases specified in the figure. Defect formation energies of major intrinsic point defects at (c) point A (Sb-rich growth condition) and (d) point B (maximally Ca-rich and Mg-rich growth condition). The blue vertical line indicates the Fermi level position at 300 K.

In order to investigate the optimum growth conditions that can enable n-type conductivity, a systematic calculation of defect formation energies under varying chemical potentials are calculated and shown in Fig. 4. Fig. 4a shows a 3 × 3 × 2 supercell of CaMg_2_Sb_2_ used for defect calculations. The formation energy of a point defect *D* in charge state *q* is calculated by:^38^1
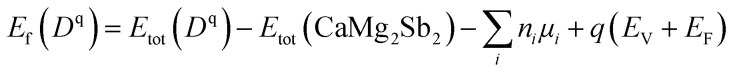
where *E*_tot_(*D*^*q*^) and *E*_tot_(CaMg_2_Sb_2_) are the total energies of the supercell with and without a defect *D* in charge state *q*, respectively. *µ*_*i*_ represents the chemical potential of the constituent *i* and *n*_*i*_ denotes the number of atoms of element *i* that has been added to (*n*_*i*_ > 0) or removed from (*n*_*i*_ < 0) the perfect supercell. *E*_V_ represents the energy at valence band maximum (VBM) of defect free supercell, and *E*_F_ is the Fermi level referenced to VBM. The calculated *E*_tot_(CaMg_2_Sb_2_) and *E*_V_ are −349.58 eV and 3.48 eV, respectively.

According to the formula *µ*_*i*_ = *µ*^0^_*i*_ + Δ*µ*_*i*_, the elemental chemical potential *µ*_*i*_ can be expressed as the sum of its standard reference value (*µ*^0^_*i*_) and a deviation (Δ*µ*_*i*_). The allowed Δ*µ*_*i*_ is limited by two requirements: maintaining the stability of the host compound and advoiding the formation of all other competing phases. Based on equilibrium conditions for the crystal growth, to maintain a stable compound, the sum of chemical potentials of its constituent atoms must equal the enthalpy of formation of the compound, which is described by:

2 Δ*µ*_Ca_ + 2Δ*µ*_Mg_ + 2Δ*µ*_Sb_ = Δ*H*_f_(CaMg_2_Sb_2_)

The formation enthalpy of CaMg_2_Sb_2_ is calculated to be −0.87 eV per atom by using the HSE06 functional. To avoid the precipitation of elemental solids, the chemical potential must be smaller than that of the corresponding elemental solid, which can be defined as:

3 Δ*µ*_Ca_ ≤ 0; Δ*µ*_Mg_ ≤ 0; Δ*µ*_Sb_ ≤ 0

The chemical potentials are further limited by requiring that other possible competing phases containing Ca, Mg, and Sb do not form. For example, if Ca and Sb naturally form Ca_2_Sb, the following condition must be satisfied:

4 2Δ*µ*_Ca_ + Δ*µ*_Sb_ ≤ Δ*H*_f_(Ca_2_Sb)

The formation enthalpy of Ca_2_Sb is calculated to be −1.02 eV per atom by using the HSE06 functional. Other possible competing phases, including CaMg_2_, Mg_3_Sb_2_, CaSb_2_, and Ca_5_Sb_3_, are also taken into account. Based on the above limiting conditions, Fig. 4b shows the computed chemical potential domains in Δ*µ*_Mg_ and Δ*µ*_Ca_ plane. The shaded regions correspond to the stability range where CaMg_2_Sb_2_ can exist. In the white regions, the CaMg_2_Sb_2_ are unstable with respect to the competing phases shown in the figure. For example, the white area on the left is excluded due to the precipitation of Mg_3_Sb_2_, whereas the white areas on the right are excluded due to the formation of CaMg_2_, Ca_2_Sb, CaSb_2_, and Ca_5_Sb_3_.

In the allowed region of Fig. 4b, point A corresponds to the Sb-rich growth condition with Δ*µ*_Sb_ = 0 eV, Δ*µ*_Ca_ = −2.97 eV and Δ*µ*_Mg_ = −0.69 eV, whereas point B corresponds to maximally Ca-rich and Mg-rich growth conditions with Δ*µ*_Sb_ = −1.82 eV, Δ*µ*_Ca_ = −0.7 eV and Δ*µ*_Mg_ = 0 eV. The formation energies of various major intrinsic defects under these two growth conditions are calculated and are presented in Fig. 4c and d. The charge state of each defect can be identified from the slope of the curve, where a positive slope indicates a donor defect and a negative slope corresponds to an acceptor defect. A defect is more favorable to form when its formation energy is more negative. As shown in Fig. 4c, under Sb-rich growth condition, the acceptor Ca vacancy (*V*_Ca_) is easier to form than the donor Mg and Ca interstitials (Mg_i_ and Ca_i_). In contrast, as shown in Fig. 4d, under maximally Ca-rich and Mg-rich growth conditions, the formation energies of Mg and Ca interstitials (Mg_i_ and Ca_i_) are lower than those of vacancies (*V*_Ca_ and *V*_Mg_), suggesting that interstitials are more likely to form under these growth conditions.

**Fig. 5:**
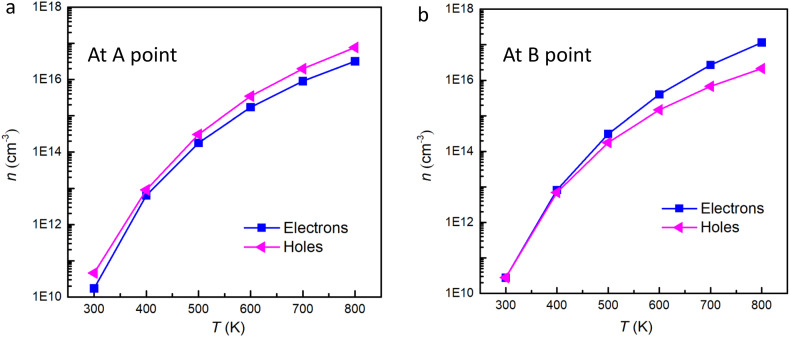
Temperature dependence of carrier concentrations at (a) point A (Sb-rich growth condition) and (b) point B (maximally Ca-rich and Mg-rich growth condition) in CaMg_2_Sb_2_ system. Pink and blue lines represent the hole and electron carrier concentrations, respectively.

The electron and holes concentration can be calculated based on charge neutrality condition,^39^ and the computational details are presented in the SI. Fig. 5 displays the predicted temperature dependence of carrier concentrations of CaMg_2_Sb_2_ system, respectively at “point A” and “point B” growth condition. Under Sb-rich growth condition, the hole concentration is larger than the electron concentration in the temperature range from 300 K to 800 K, which is mainly because Ca vacancies are the dominant point defects. At 725 K, the hole and electron concentrations are about 2 × 10^16^ cm^−3^ and 9.1 × 10^15^ cm^−3^, respectively, yielding a net carrier concentration of 1.09 × 10^16^ cm^−3^, which corresponds to a *zT* value of 3.9 × 10^−4^ (Fig. 3d). However, under maximally Ca-rich and Mg-rich growth conditions, the electron concentration exceeds the hole concentration in the medium-to-high temperature range, primarily due to Mg interstitials and Ca interstitials being the dominant point defects. At 725 K, the electron and hole concentrations are about 3 × 10^16^ cm^−3^ and 7 × 10^15^ cm^−3^, respectively, yielding a net carrier concentration of 2.3 × 10^16^ cm^−3^, which corresponds to a *zT* value of 3 × 10^−3^ (Fig. 3d). Appropriate donor doping can be expected to further substantially increase the electron concentration, thus optimizing the thermoelectric performance. From the above analysis, it can be inferred that the condition favorable for achieving n-type transport in CaMg_2_Sb_2_ is to establish Ca-rich and Mg-rich growth conditions.

## 4. Conclusions

In this work, the defect chemistry, electronic structure, and thermoelectric transport properties of CaMg_2_Sb_2_ are systematically investigated using first-principles calculations. The compound exhibits an indirect band gap of 1.07 eV. Electrical transport simulations reveal that n-type compound achieves a much higher power factor than its p-type counterpart, with a maximum power factor of 59 µW cm^−1^ K^−1^ at 300 K and 39 µW cm^−1^ K^−1^ at 725 K at a carrier concentration of 1 × 10^20^ cm^−3^. Combining the simulated lattice and electronic thermal conductivity, the n-type system exhibits a high thermoelectric Figure of Merit, reaching a maximum *zT* of approximately 1.52 at 725 K with a carrier concentration of ∼1 × 10^20^ cm^−3^.

Defect analysis reveals that under Sb-rich growth conditions, the acceptor Ca vacancy is the dominant point defect, leading to p-type conduction. In contrast, under maximally Ca-rich and Mg-rich growth conditions, donor Mg and Ca interstitials become more favorable, which results in n-type conduction with the electron concentration of 3 × 10^16^ cm^−3^ at 725 K. Therefore, establishing Mg-rich and Ca-rich growth conditions is a key factor for achieving the high-performance n-type thermoelectric transports in CaMg_2_Sb_2_. This study offers theoretical insights for designing high-performance n-type AM_2_X_2_ thermoelectric materials.

## Conflicts of interest

There are no conflicts of interest to declare.

## Supplementary Material

RA-016-D6RA03855G-s001

## Data Availability

All data needed to evaluate the conclusions of this study are included in the article. Any additional information related to this study may be requested from the authors. Supplementary information (SI): calculation details of the relaxation time, minimum lattice thermal conductivity, and electron and hole concentrations. See DOI: https://doi.org/10.1039/d6ra03855g.
