# Supplementary material for: First-principles study of defect chemistry and thermoelectric performance of CaMg2Sb2
Source: RSC Adv. 2026 Jul 2;16(34):32632–9. doi: 10.1039/d6ra03855g (PMC13325527; doi:10.1039/d6ra03855g)
Supplement: RA-016-D6RA03855G-s001 [file RA-016-D6RA03855G-s001.pdf]

Supplementary Information (SI) for RSC Advances.
This journal is © The Royal Society of Chemistry 2026

Supporting Information

First-principles study of defect chemistry and thermoelectric performance of $CaMg_2Sb_2$ compound

Shuai Zhang,[a,b] Yuan Liu,[a] Wenjing Qiu,[a] Juan Li,[a,*] Qidong Wang,[a,c] Kai Han,[a] Xiaole Qiu[a] and Bing Sun[a,**]

[a]School of Physics and Electronic Information, Shandong Key Laboratory of Gallium Nitride Materials and Applications, Weifang University, Weifang 261061, China

[b]Jinan Key Laboratory of X-ray Optics, Jinan 250000, China

[c]Key Laboratory of Low-Dimensional Structural Physics and Application, Education Department of Guangxi Zhuang Autonomous Region, College of Physics and Electronic Information Engineering, Guilin University of Technology, Guilin 541004, China

Corresponding authors:

[*]Emails: lj_wfu@163.com (Juan Li); 20170005@wfu.edu.cn (Bing Sun)

Deformation potential theory

The relaxation time (τ) is estimated by using the deformation potential theory，which is expressed as:[1]

$$\tau = \frac{2\sqrt{2\pi}\hbar^4 c_{ii}}{3(k_B T m_s^*)^{3/2}\Xi^2} \quad (1)$$

where c_{ii} is the lattice elastic constant, m_s^* is the single valley effective mass and Ξ is the deformation potential. The lattice elastic constants c_{11}, c_{22} and c_{33} are 81.7 GPa, 81.7 GPa and 67.1 GPa, leading to the average value of 76.8 GPa. The deformation potential constant is estimated by the formula, $\Xi = \partial E_{edge} / \partial(\Delta a/a)$, where E_{edge} is the energy of conduction band maximum (CBM)/valence band minimum (VBM), a is the lattice constant and Δa, given by $\Delta a = a - a_0$, is the corresponding lattice distortion.

Calculation of the minimum lattice thermal conductivity

The minimum lattice thermal conductivity $\kappa_{\min}$ is calculated using the Cahill's formula:[2,3]

$$\kappa_{\min} = \frac{1}{2}[(\frac{\pi}{6})^{1/3}]k_B (V)^{-2/3}(2v_t + v_l) \quad (2)$$

where V is the average volume per atom, v_t is traverse elastic wave velocity and v_l is longitudinal elastic wave velocity. v_t and v_l can be obtained using the following equations:

$$v_t = \sqrt{\frac{G}{\rho}} \quad (3)$$

$$v_l = \sqrt{(B + \frac{4}{3}G)/\rho} \quad (4)$$

where B is the bulk modulus, G is the shear modulus and ρ is the density. B and G are determined adopting the Voigt-Reuss-Hill averaging scheme in hexagonal system given by:[4,5]

$$B = (B_V + B_R)/2 \tag{5}$$

$$G = (G_V + G_R)/2 \tag{6}$$

$$B_V = \frac{1}{9}(2(c_{11} + c_{12}) + 4c_{13} + c_{33}) \tag{7}$$

$$B_R = \frac{c^2}{M} \tag{8}$$

$$G_V = \frac{1}{30}(M + 12c_{44} + 12c_{66}) \tag{9}$$

$$G_R = \frac{5c^2 c_{44} c_{66}}{2(3B_V c_{44} c_{66} + c^2(c_{44} + c_{66}))} \tag{10}$$

$$M = c_{11} + c_{12} + 2c_{33} - 4c_{13} \tag{11}$$

$$c^2 = (c_{11} + c_{12})c_{33} - 2c_{13}^2 \tag{12}$$

where c_{11}, c_{33}, c_{44}, c_{12} and c_{13} are five independent elastic constants for hexagonal system and are estimated to be 81.7 GPa, 67.1 GPa, 25.9 GPa, 30 GPa and 21.5 GPa, respectively.

Estimation of electron and holes concentration

The electron and holes concentrations are calculated based on charge neutrality condition, which can be expressed by:[6]

$$\sum_i q_i c_i(D^q) + p - n = 0 \tag{13}$$

where index i represents the charged defect, c_i denotes the concentration of a point defect D with charge state q, and n and p are the free carrier concentrations of electrons and holes. The c_i, n and p are determined by the following equations:

$$c(D^q) = N_{\mathrm{site}}\, exp\left(-\frac{E_f(D^q)}{k_{\mathrm{B}}T}\right) \tag{14}$$

$$n = \int_{E_{\mathrm{C}}}^{+\infty} g(E) f(E; E_{\mathrm{F}}, T)\mathrm{d}E \tag{15}$$

$$p = \int_{-\infty}^{E_{\mathrm{V}}} g(E)[1 - f(E; E_{\mathrm{F}}, T)]\mathrm{d}E \tag{16}$$

where N_{site} is the concentration of possible defect sites, k_{B} is the Boltzmann constant, T is the absolute temperature, E_{V} is valence band maximum, E_{C} is the conduction band minimum, $g(E)$ is the density of states of the ideal host cell, and $f(E; E_{\mathrm{F}}, T)$ is the Fermi-Dirac distribution, which is expressed by:

$$. \ f(E; E_{\mathrm{F}}, T) = \frac{1}{exp\left(\frac{E - E_{\mathrm{F}}}{k_{\mathrm{B}}T}\right) + 1} \tag{17}$$

References

1. J. Bardeen and W. Shockley, *Phys. Rev.,* 1950, **80**, 72.

2. D. G. Cahill, S. K. Watson and P. Ro, *Phys. Rev. B*, 1992, **46**, 6131-6140.

3. T. Fang, S. Zheng, H. Chen, H. Cheng, L. Wang and P. Zhang, *RSC Adv.*, 2016, **6**, 10507-10512.

4. Z. J. Wu, E. J. Zhao, H. P. Xiang, X. F. Hao, X. J. Liu and J. Meng, *Phys. Rev. B*, 2007, **76**, 054115.

5. J. I. Tani, M. Takahashi and H. Kido, *Physica B*, 2010, **405**, 4219.

6. X. Chong, P.-W. Guan, Y. Wang, S.-L. Shang, J. P. S. Palma, F. Drymiotis, V. A. Ravi, K. E. Star, J.-P. Fleurial and Z.-K. Liu, *ACS Appl. Energy Mater.,* 2018, **1**, 6600-6608.
